# Supplementary material for: A novel hybrid framework based on temporal convolution network and transformer for network traffic prediction
Source: PLoS One. 2023 Sep 8;18(9):e0288935. doi: 10.1371/journal.pone.0288935 (PMC10490908; doi:10.1371/journal.pone.0288935)
Supplement: Attachment — Submitted filename: response_letter_plosone.pdf [file pone.0288935.s001.pdf]

Paper title: A Novel Hybrid Framework Based on Temporal Convolution Network and Transformer for Network Traffic Prediction

Manuscript ID: PONE-D-22-32685

Dear Prof. Son,

Thank you for giving us a chance to revise our manuscript. We believe we have fully addressed the reviewer's comments by making a revision (the revised content is labelled in red). We have updated the introduction with many recent research results on the topic of time series forecasting to better reflect the cutting-edge and innovative nature of the research. Moreover, we have also removed the preprint papers. We sincerely apologise for the lack of figures in the previous manuscript and have inserted figures in the current version. We have also added a table to explain the temporal and spatial features of the data in 3.1.1. We have also explained the method of comparison between models in the experimental section, explaining the meaning and calculation of data such as "50.79%", "64.97%", etc. A list of point-to-point responses with respect to the reviewers' comments is given below. Should you have any questions, feel free to contact us.

Yours sincerely,
Zhiwei Zhang, Shuhui Gong, Zhaoyu Liu, Da Chen.

To Reviewer 1:
First of all, thanks for your patient reading and kind suggestions. In the revised version, we have updated the references in the introduction, removed preprint papers, inserted figures on the paper, added tables to explain the features of the data, and also explained the calculation of the data such as "50.79% and 64.97%" in 3.3.

Below we give our point-to-point responses with respect to the comments.

Comment 1: "References are out-up-date. Please update them. Also please remove several preprint papers. The authors should update the introduction section with up-to-date studies on Time series prediction topic"
Response: We appreciate the reviewer pointing out our outdated references, citing preprint papers, and other issues. In the current version, we have removed the outdated papers (reference 4 and 7 in the previous manuscript) and updated it with the latest research results. We also have removed the preprint papers in the latest manuscript (reference 10, 16, 17 and 25 in the previous manuscript).

We also added references in the revised manuscript, which are shown below:

"*Tian et al. used the ARMA model to predict the network traffic and optimized the prediction results using an optimization function. Moreover, its performance is improved [4].* " **(Page 1, Line 44-46)**
"*Li et al. proposed a smoothing-aided support vector machine(SSVM) model, and this model showed superior nonlinear approximation capacity and outperformed the baseline model. [7].* " **(Page 2, Line 55-57)**
"*Jiang used deep learning models to predict traffic compared to 5 baseline models, and all deep learning models outperformed the baseline models, especially InceptionTime, which had the most accurate predictions [10].* " **(Page 2, Line 63-65)**
"*Wan et al. proposed a neural network model based on Long Short-Term Memory (LSTM) and transfer learning and applied it to the network traffic prediction problem. Compared with the direct training model without transfer learning, the performance was improved by 40% [11].* " **(Page 2, Line 66-68)**
"*However, the Temporal Convolutional Network (TCN) can better extract temporal information features [17], and then the convolutional layer in TCN was extended to three dimensions to extract spatial information features in the data.*" **(Page 2, Line 85-87)**
"*First, we used TCN based on the Convolutional Block Attention Module (CBAM) attention mechanism [18] to extract spatio-temporal local features and reduce the size of the data.*" **(Page 2, Line 88-90)**
"*This function is somewhat similar to the mean square error (MSE) formula and has all the advantages of the Huber loss function [26].*" **(Page 11, Line 272-274)**

“*4.Tian M, Sun C, Wu S. An EMD and ARMA-based network traffic prediction approach in SDN-based internet of vehicles. Wireless Networks, 2021: 1-13.*” **(Page 16, Line 423-424)**

“*7.Li Y, Wang J, Sun X, et al. Smoothing-aided support vector machine based nonstationary video traffic prediction towards B5G networks. IEEE Transactions on Vehicular Technology, 2020, 69(7): 7493-7502.*” **(Page 16, Line 432-434)**

“*10.Jiang W. Internet traffic prediction with deep neural networks. Internet Technology Letters, 2022, 5(2): e314.*” **(Page 16, Line 440-441)**

“*11.Wan X, Liu H, Xu H, et al. Network Traffic Prediction Based on LSTM and Transfer Learning. IEEE Access, 2022, 10: 86181-86190.*” **(Page 16, Line 442-443)**

“*17. Fan J, Zhang K, Huang Y, et al. Parallel spatio-temporal attention-based TCN for multivariate time series prediction. Neural Computing and Applications, 2021: 1-10.*” **(Page 17, Line 457-458)**

“*18. Woo S, Park J, Lee J Y, et al. Cbam: Convolutional block attention module. Proceedings of the European conference on computer vision (ECCV). 2018: 3-19.*” **(Page 17, Line 459-460)**

“*26. Jadon S. A survey of loss functions for semantic segmentation. 2020 IEEE conference on computational intelligence in bioinformatics and computational biology (CIBCB). IEEE, 2020: 1-7.*” **(Page 17, Line 479-481)**

Comment 2: “The authors should insert the images on the paper. The current version is so difficult to follow.”

Response: We apologised sincerely for this. In the previous version, we have inserted images in the previous manuscript. However, it may not appear in the previous version.

The images in the manuscript are shown below:

1.

Fig 1. Pearson heat map of the network traffic flow data of the Milan city network

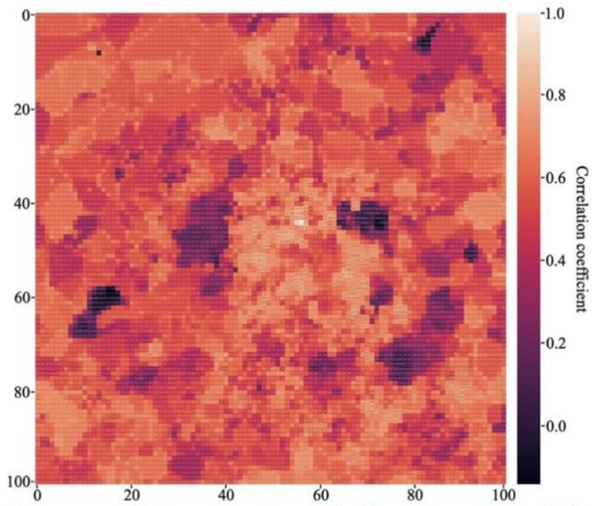

Fig 1. Pearson heat map of the network traffic flow data of the Milan city network

(in Section 2, Page 4)

2.

Fig 2. Relationship between models

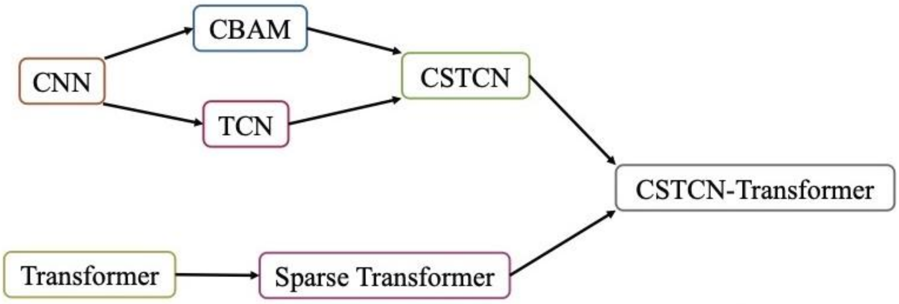

Fig 2. Relationship between models

(in Section 2, Page 4)

3.

Fig 3. Schematic diagram of a CNN

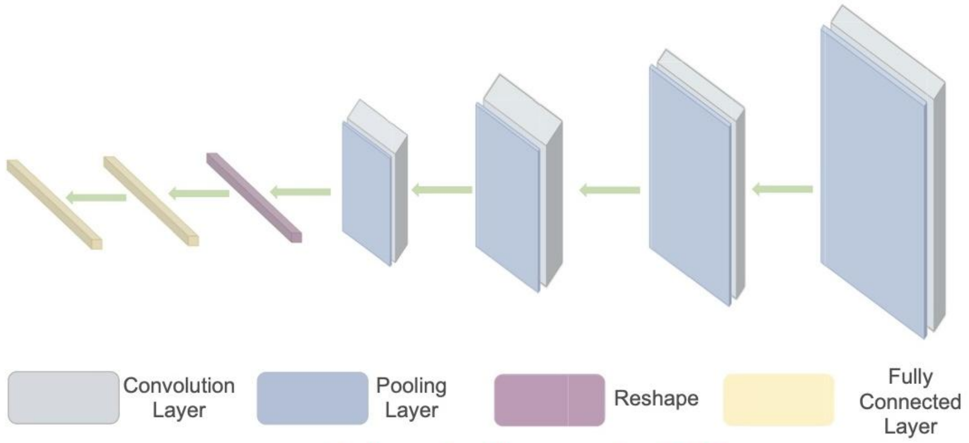

Fig 3. Schematic diagram of a CNN

(in Section 2, Page 5)

4.
Fig 4. Schematic diagram of the causal convolution structure

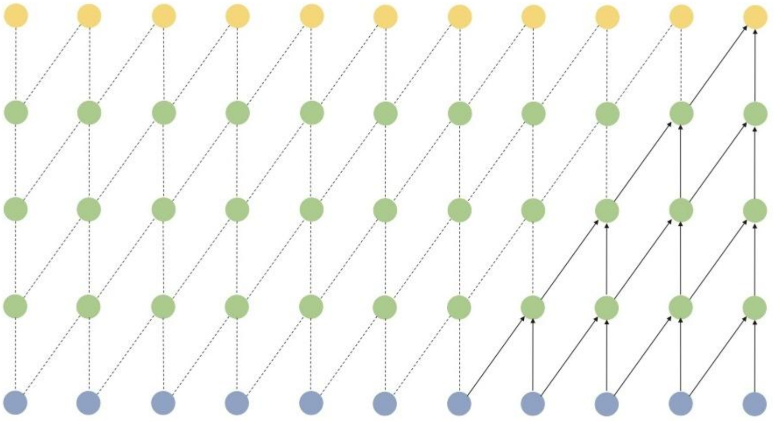

Fig 4. Schematic diagram of the causal convolution structure

(in Section 2, Page 5)

5.
Fig 5. Schematic diagram of the expansion convolution structure

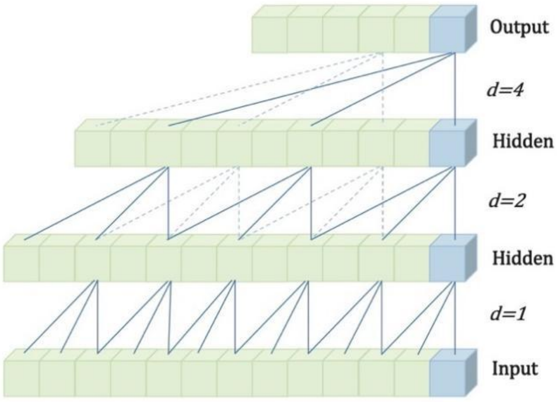

Fig 5. Schematic diagram of the expansion convolution structure

(in Section 2, Page 6)

6.

Fig 6. Schematic diagram of the TCN residual module

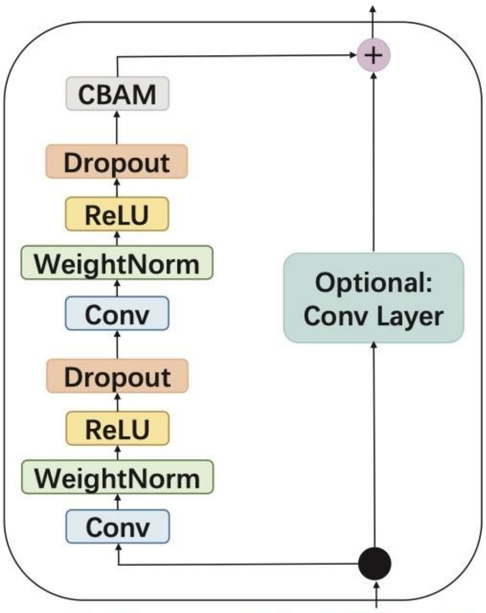

Fig 6. Schematic diagram of the TCN residual module

(in Section 2, Page 6)

7.

Fig 7. Schematic diagram of the CBAM block structure

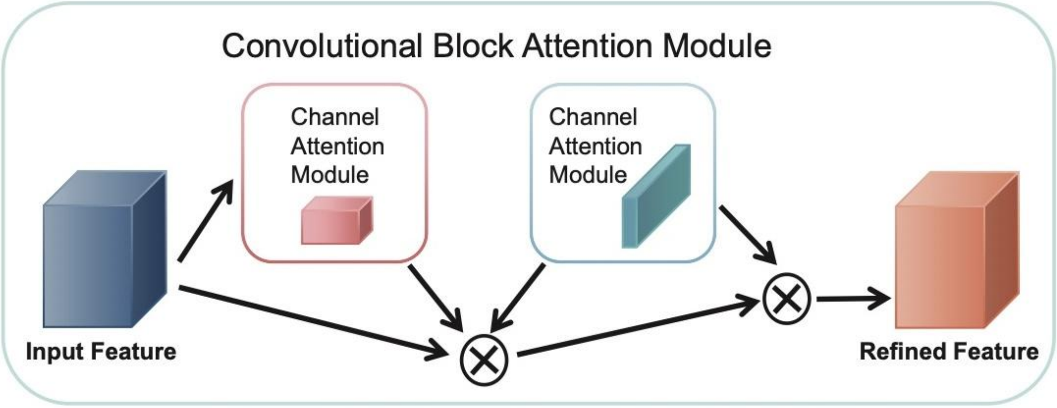

Fig 7. Schematic diagram of the CBAM block structure

(in Section 2, Page 7)

8.
Fig 8. Schematic diagram of the CAM module structure

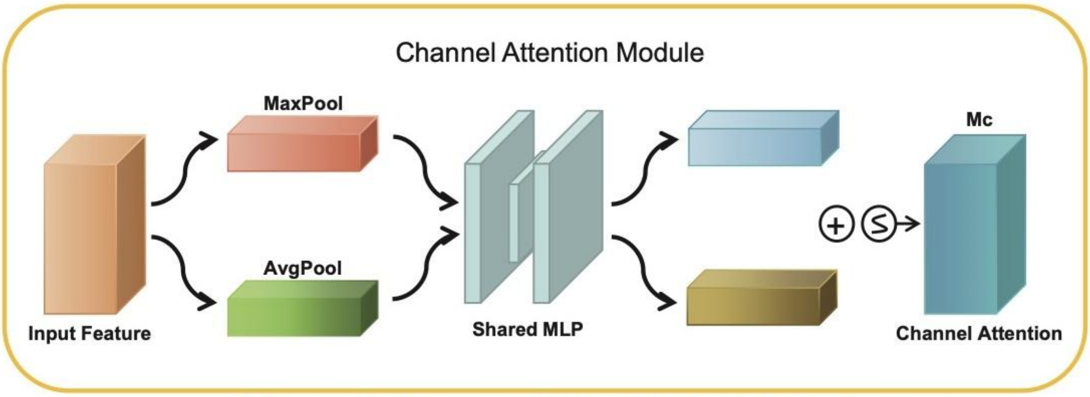

Fig 8. Schematic diagram of the CAM module structure

(in Section 2, Page 7)

9.
Fig 9. Schematic diagram of the SAM module structure

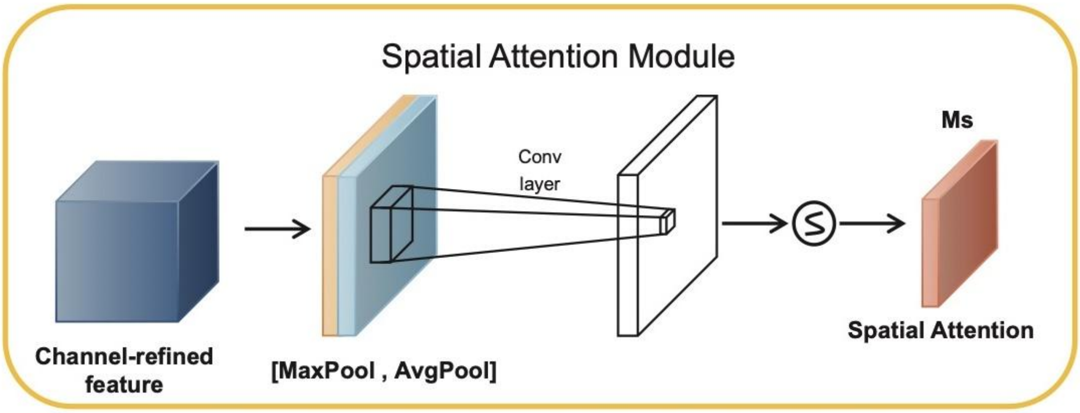

Fig 9. Schematic diagram of the SAM module structure

(in Section 2, Page 8)

10.

Fig 10. Schematic diagram of the Transformer structure

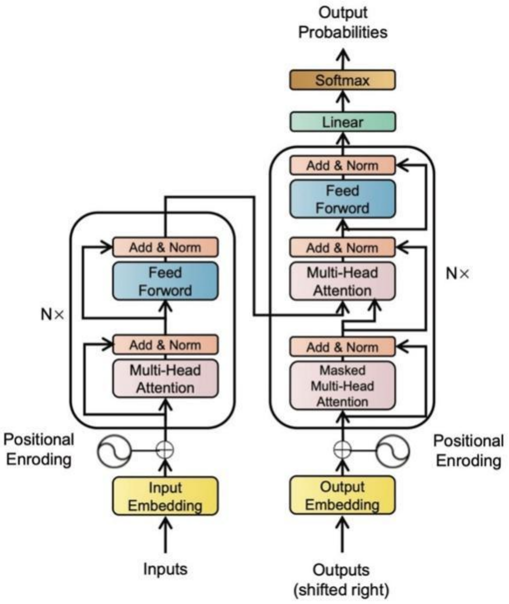

Fig 10. Schematic diagram of the Transformer structure

(in Section 2, Page 9)

11.

Fig 11. Schematic diagram of the Transformer self-attention mechanism

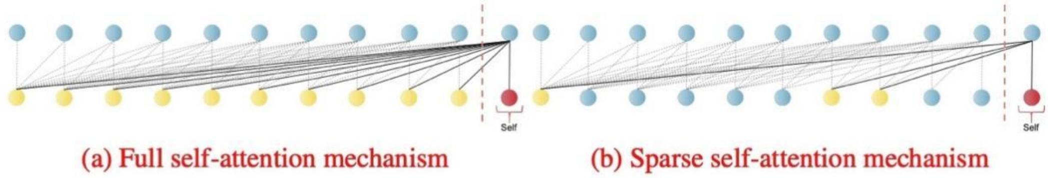

Fig 11. Schematic diagram of the Transformer self-attention mechanism

(in Section 2, Page 10)

12.

Fig 12. Overall structure of the CSTCN-Transformer

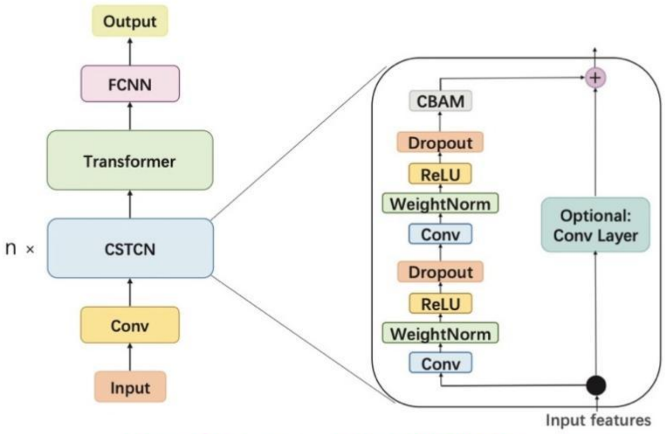

Fig 12. Overall structure of the CSTCN-Transformer

(in Section 2, Page 10)

13.

Fig 13. Area division map of Milan

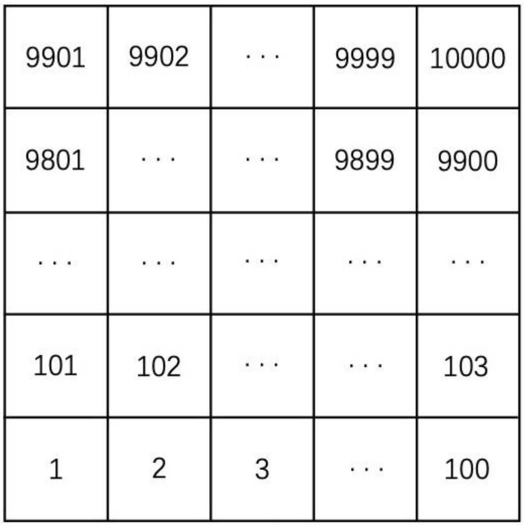

Fig 13. Area division map of Milan

(in Section 3, Page 11)

14.

Fig 14. Prediction effect of CSTCN-Transformer

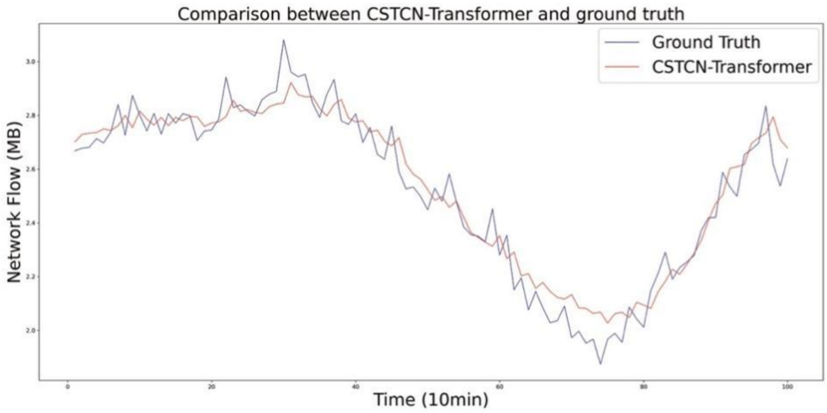

Fig 14. Prediction effect of CSTCN-Transformer

(in Section 3, Page 14)

Comment 3: “In 3.1.1, the authors should add a table that show the feature name and feature description.”

Response: We thank the reviewer for suggesting adding a table to describe the features. We added a table for describing temporal features and spatial features in 3.1.1 and introduced data size, range, and minimum unit for these two features in the table.

This is the effect in the revised manuscript:

In summary, the dataset contains both temporal and spatial information and its size is 4320×100×100. Its spatio-temporal features are presented in Tables 1.

Table 1. Spatio-temporal Features of Dataset

Feature name	Data size	Range	Minimum unit
Temporal features	4320	Nov. 1, 2013 — Nov. 30, 2013	Every 10 minutes
Spatial features	100×100	10,000 grids of the city of Milan	Network traffic per grid

(Page 11, Line 293-294)

Comment 4: “in the experiment section, please explain the numbers “50.79% and 64.97%” are come from? How to calculate them?”

Response: We appreciate the reviewer's suggestion that the manuscript was lacking in calculating the percentage data. We have added specific formulas and instructions for percentage data, such as "50.79% and 64.97%" in 3.3.

This is the effect in the revised manuscript:

We use the percentage calculation method to assess the evaluation criteria for model prediction accuracy with the following equation:

$$p = \frac{b - a}{b} \times 100\%, \tag{11}$$

where b is larger than a and p denotes the percentage decrease of a relative to b.

(Page 14, Line 350-352)
