# Supplementary material for: A novel hybrid framework based on temporal convolution network and transformer for network traffic prediction
Source: PLoS One. 2023 Sep 8;18(9):e0288935. doi: 10.1371/journal.pone.0288935 (PMC10490908; doi:10.1371/journal.pone.0288935)
Supplement: Attachment — Submitted filename: response_letter_june_19.pdf [file pone.0288935.s002.pdf]

Paper title: A novel hybrid framework based on temporal convolution network and transformer for network traffic prediction

Manuscript ID: PONE-D-22-32685R1

Dear Prof. Le Hoang Son

Thank you for giving us a chance to revise our manuscript. We believe that we have fully addressed the reviewers' comments by revising the experiments of our paper (the revised content is labelled in red).

In particular, we added comparisons with the latest baseline model, conducted experiments to measure long-term performance, and added MAPE as an evaluation metric. Although we had the ablation experiments before, we now separated the comparison between the ablation experiments and the baseline model to make it clearer. We also provided detailed explanations of the model's shortcomings, modified the literature review, and addressed other issues raised by the reviewers. A list of point-to-point responses concerning the reviewers' comments is given below. Should you have any questions, feel free to contact us.

Yours sincerely,

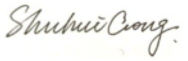

To Reviewer 1:

Comment: "All comments are addressed. Therefore, I suggest accept this manuscript."

We thank the reviewer for accepting our manuscript.

To Reviewer 2:

First of all, thanks for your patient reading and kind suggestions. In the revised manuscript, We added a new baseline model, modified the presentation of the ablation experiments, added MAPE as another evaluation metric, and also tested the model's long-term prediction performance. We also created a bar chart that not only shows the contribution of the attention mechanism but also allows for a better comparison of the effects of other modules in the model and its performance compared to numerous baseline models. We used FLOPs and Params to represent the spatial and temporal complexity of the model and pointed out its shortcomings. We modified the literature review by describing the development process of the attention mechanism and explaining how the referenced literature improved upon previous work. We also explained the conditions for using Pearson correlation coefficients and made modifications to the images to improve readability. Finally, we corrected errors such as italics and boldface in the article.

Below we give our point-to-point responses concerning the comments.

Major comments:

Comment 1: "The authors should use floating point of operations (FLOPs) and Params to represent spatial-temporal complexity."

Response: Thank you for raising this question. The FLOPs of the model are 3.19 M, and 916.88 M respectively. We also provided the related explanations in the revised manuscript:

this paper, we used a batch size of 128, an adam optimizer, a learning rate of 0.001, and ıt rate of 0.02. We used 80% of the data as the training set and 20% of the data as the et. The parameters and FLOPs of this model are 3.19M and 916.88M respectively. es 2—6 show the specific hyperparameters of the model proposed in this paper.

(Page 14, Line 393)

Comment 2: "The authors have to elaborate on the shortcomings of the model."

Response: Thank you for raising this question. In our view, the model is somewhat large, which could lead to increased training and operational costs or some challenges in tuning hyperparameters.

We also provided the related explanations in the revised manuscript:

in this paper can also be applied in the task of predicting such data.

Limitation: The integration of TCN, CBAM, and Sparse-Transformer may lead to high complexity, slowing down training and inference. This could impact performance without a GPU or with limited GPU memory. Hyperparameter tuning: The model involves multiple network structures and numerous hyperparameters. Finding the optimal combination may be time-consuming and resource-intensive.

Our future work will consider using the prediction results of the model in this paper to

(Page 19, Line 480)

Comment 3(1): "Ablation experiments need to be added to clarify the role of each module."

Response: Thank you for raising this question. Although the article originally included ablation experiments using the TCN, CBTCN, CSTCN, Transformer, Sparse-Transformer, and Adding redundant information models, it was not explicitly stated that these were ablation experiments. We have now separated the ablation experiments from the baseline models proposed by others for greater clarity and readability. The ablation experiment results can be found on Page 17, Table 8 and Page 17, Fig 16, and these sentences have been highlighted in red font.

We also provided the related explanations in the revised manuscript:

Table 8. Performance Comparison with Ablation Experiment Models

Model	MSE	MAE	MAPE
TCN	2.45×10^{-2}	1.69×10^{-1}	4.69×10^{-2}
CBTCN	1.91×10^{-2}	1.44×10^{-1}	4.40×10^{-2}
CSTCN	1.78×10^{-2}	1.29×10^{-1}	4.07×10^{-2}
Transformer	1.56×10^{-2}	1.02×10^{-1}	3.93×10^{-2}
Sparse-Transformer	1.26×10^{-2}	9.43×10^{-2}	3.57×10^{-2}
CSTCN-Transformer	6.21×10^{-3}	6.28×10^{-2}	2.51×10^{-2}
Adding redundant information	6.65×10^{-3}	6.53×10^{-2}	2.64×10^{-2}

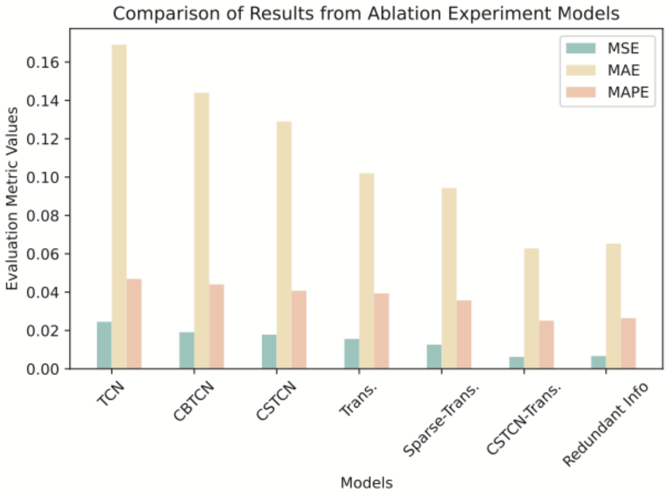

Fig 16. Comparison of results from ablation experiment models

By observing the Fig. 16 and Table 8, the following conclusion can be obtained:

First, compared to TCN, CBTCN reduced MSE, MAE, and MAPE by 22.04%, 14.62%, and 6.18%, respectively, demonstrating that the introduction of the CBAM attention mechanism can help the model better focus on useful information, thus improving prediction accuracy. Second, in the comparison between Transformer and Sparse-Transformer, Sparse-Transformer decreased MSE, MAE, and MAPE by 19.23%, 23.53%, and 9.16%, respectively, indicating that the sparse

self-attention mechanism can enhance the accuracy of network traffic prediction results and prevent overfitting.

Furthermore, compared to CSTCN, the CSTCN-Transformer reduced MSE, MAE, and MAPE by 65.17%, 51.16%, and 38.33%, respectively, suggesting that the Transformer's self-attention mechanism can help the model extract spatiotemporal features more effectively. Lastly, compared to data with redundant information, the CSTCN-Transformer's MSE, MAE, and MAPE for processing normal data decreased by only 6.65%, 3.83%, and 4.92%, further proving the superior effects of CBAM and Transformer attention, even when dealing with a large amount of redundant information.

In the comparison between Sparse-Transformer and CSTCN-Transformer, CSTCN-Transformer exhibited higher prediction accuracy, reducing MSE, MAE, and MAPE by 50.79%, 33.40%, and 29.69%, respectively. This is attributed to the introduction of CSTCN, which combines spatiotemporal convolutional networks and CBAM attention mechanisms, enabling the model to more effectively extract spatiotemporal features and focus on useful information.

[illegible]

(Page 17, Line 419-442)

Comment 3(2): "Comparison of the efficiency of the proposed model with some latest models should be added."

Response: Thank you for raising this question. We have added the recently popular GRU, InceptionTime, and ResNet models as baseline models to compare with our experimental results. The results of the baseline model experiments can be found in Table 7 and Fig 15, and these sentences have been highlighted in red font.

We also provided the related explanations in the revised manuscript:

Table 7. Performance Comparison with Baseline Models

Model	MSE	MAE	MAPE
LSTM	1.77×10^{-2}	1.34×10^{-1}	4.16×10^{-2}
GRU	1.58×10^{-2}	1.25×10^{-1}	4.01×10^{-2}
InceptionTime	1.29×10^{-2}	1.01×10^{-1}	3.89×10^{-2}
ResNet	1.36×10^{-2}	1.11×10^{-1}	4.04×10^{-2}
CSTCN-Transformer	6.21×10^{-3}	6.28×10^{-2}	2.51×10^{-2}

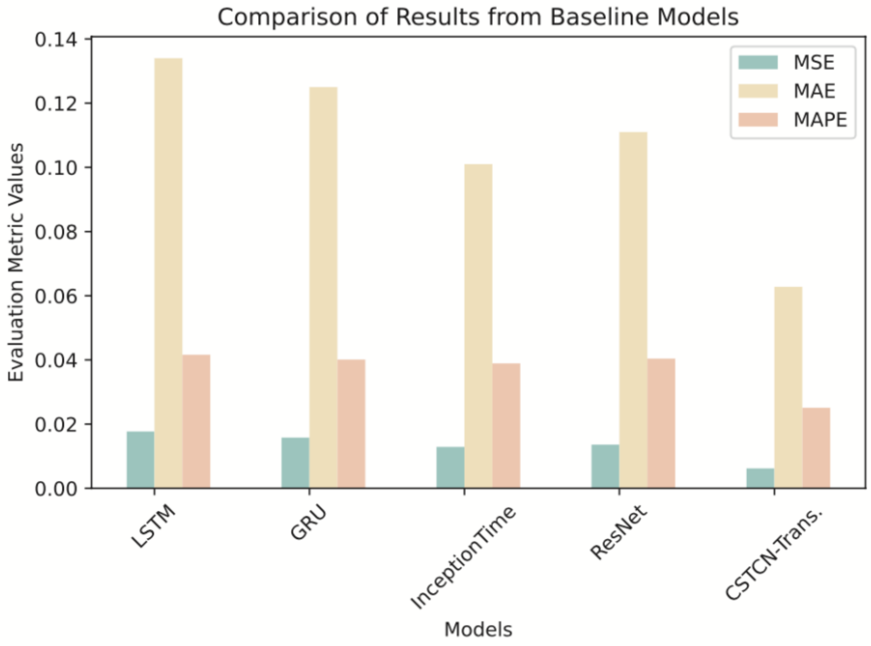

Fig 15. Comparison of results from baseline models

(Page 16, Table 7 and Fig. 15)

The Fig. 15 and Table 7 show that the CSTCN-Transformer significantly outperforms other models in terms of MSE, MAE, and MAPE. Compared to LSTM, GRU, InceptionTime, and ResNet, the CSTCN-Transformer reduces MSE, MAE, and MAPE by 64.91%, 60.76%, 51.94%, and 54.34%; 53.10%, 49.60%, 37.62%, and 43.42%; and 39.66%, 37.41%, 35.47%, and 37.87%, respectively, indicating that the combination of Sparse-Transformer, CBAM, and TCN can indeed better extract useful information and achieve more accurate results.

Among LSTM, GRU, InceptionTime, and ResNet, InceptionTime performs better in MSE, MAE, and MAPE, with values of 1.29×10^{-2}, 1.01×10^{-1}, and 3.89×10^{-2}, respectively. Although the performance of these models in network traffic prediction tasks is not significantly different, InceptionTime shows relatively better performance among these models.

In summary, the CSTCN-Transformer demonstrates a significant advantage in mobile network traffic prediction tasks compared to LSTM, GRU, InceptionTime, and ResNet, which can be attributed to its ability to effectively extract spatiotemporal features and avoid overfitting.

(Page 17, Line 406-418)

Comment 3(3): "In experiments, it is suggested to add the Mean Absolute Percentage Error (MAPE) as another evaluation indicator."

Response: Thank you for raising this question. We have added MAPE as an evaluation metric, introduced what MAPE is, and included MAPE information in the tables. We introduced MAPE in Eq. 11 (Page 13) and added MAPE information in Table 7 (Page 16), Table 8 (Page 17), and Table 9 (Page 18). These pieces of information have been highlighted in red font.

We also provided the related explanations in the revised manuscript:

$$MAPE = \frac{1}{n}\sum_{i=1}^{n} | \frac{\hat{y}_i - y_i}{y_i} |, \quad (11)$$

where $\hat{y}$ is the predicted value and y is the true value.

(Page 13)

Table 7. Performance Comparison with Baseline Models

Model	MSE	MAE	MAPE
LSTM	1.77×10^{-2}	1.34×10^{-1}	4.16×10^{-2}
GRU	1.58×10^{-2}	1.25×10^{-1}	4.01×10^{-2}
InceptionTime	1.29×10^{-2}	1.01×10^{-1}	3.89×10^{-2}
ResNet	1.36×10^{-2}	1.11×10^{-1}	4.04×10^{-2}
CSTCN-Transformer	6.21×10^{-3}	6.28×10^{-2}	2.51×10^{-2}

(Page 16)

Table 8. Performance Comparison with Ablation Experiment Models

Model	MSE	MAE	MAPE
TCN	2.45×10^{-2}	1.69×10^{-1}	4.69×10^{-2}
CBTCN	1.91×10^{-2}	1.44×10^{-1}	4.40×10^{-2}
CSTCN	1.78×10^{-2}	1.29×10^{-1}	4.07×10^{-2}
Transformer	1.56×10^{-2}	1.02×10^{-1}	3.93×10^{-2}
Sparse-Transformer	1.26×10^{-2}	9.43×10^{-2}	3.57×10^{-2}
CSTCN-Transformer	6.21×10^{-3}	6.28×10^{-2}	2.51×10^{-2}
Adding redundant information	6.65×10^{-3}	6.53×10^{-2}	2.64×10^{-2}

(Page 17)

Table 9. Comparison of Real-time Prediction and Long-term Prediction Performance

Model	MSE	MAE	MAPE
Real-time Prediction	6.21×10^{-3}	6.28×10^{-2}	2.51×10^{-2}
Long-term Prediction	6.81×10^{-3}	6.68×10^{-2}	2.93×10^{-2}

(Page 18)

Comment 3(4): “The long-term predictive performance of the model should be measured.”

Response: Thank you for raising this question. The original intention of proposing this model was to make real-time, short-term predictions. However, to demonstrate the effectiveness of the model, we also conducted experiments on long-term predictions. These pieces of information have been highlighted in red font.

We also provided the related explanations in the revised manuscript:

As a model aimed at real-time prediction of network traffic, we wanted to ensure its full potential is utilized. Therefore, we also evaluated its long-term prediction performance with a time interval 10 times greater than the original. The results are presented in Table 9.

Table 9. Comparison of Real-time Prediction and Long-term Prediction Performance

Model	MSE	MAE	MAPE
Real-time Prediction	6.21×10^{-3}	6.28×10^{-2}	2.51×10^{-2}
Long-term Prediction	6.81×10^{-3}	6.68×10^{-2}	2.93×10^{-2}

The Table 9 shows that the long-term prediction performance is only slightly worse than the real-time prediction performance. Specifically, the MSE, MAE, and MAPE values for long-term prediction are approximately 9.65%, 6.37%, and 16.73% higher than those for real-time prediction, respectively.

This minor difference between the real-time and long-term prediction performances indicates that the model is quite robust and effective, as it maintains a relatively consistent level of accuracy across varying prediction timeframes.

(Page 18, Line 440-449)

Comment 3(5): “Can the contribution of attention mechanisms be visualized to make the ideas in the article more convincing?”

Response: We are extremely grateful for the question raised by the reviewers, which has greatly inspired us. We used bar charts to visualize the results of the ablation experiments, which better demonstrate the effectiveness of the attention mechanism. Because this visualization method is effective, we also used it in the comparison of baseline models. Specifically, we displayed it in Fig 15 and Fig 16.

We also provided the related explanations in the revised manuscript:

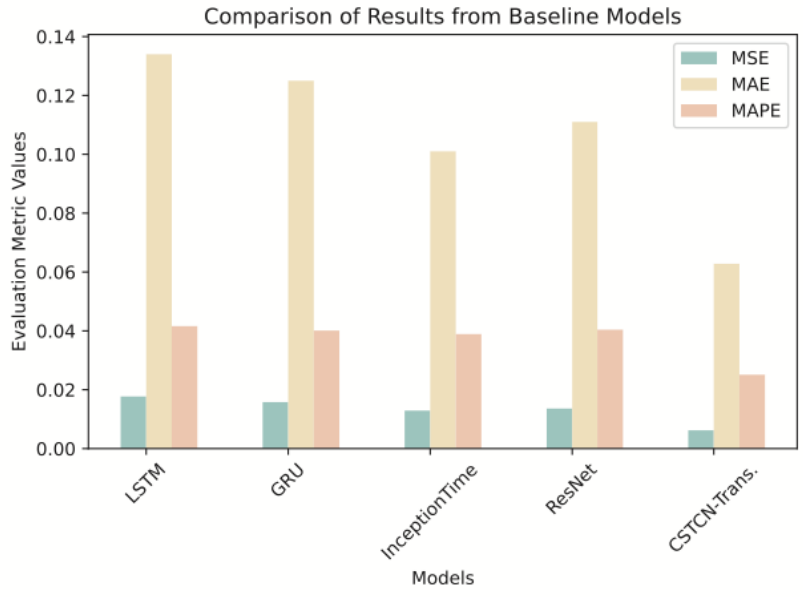

Fig 15. Comparison of results from baseline models

(Page 16)

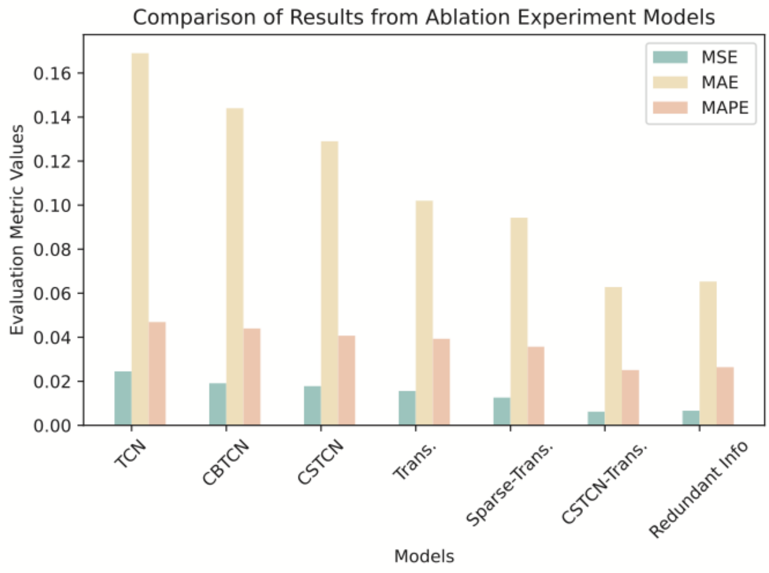

Fig 16. Comparison of results from ablation experiment models

(Page 17)

Minor comments:

Comment 1: The 1 × 1 in line 172 of the manuscript does not require italics, only variables require italics.

Response: Thank you for raising the detailed issue in the review. We have addressed this problem and made a total of 9 modifications throughout the manuscript.

We also provided the related explanations in the revised manuscript :

residual to be learned. A residual block that used a 1×1 convolutional kernel was also introduced into the STCN, as shown in Fig. 6. A dropout mechanism was also added to the STCN after each

(Page 7, line 202)

convolutional layers did not match, there was a problem in processing the data. In this case, an optional convolutional layer with a kernel size of 1×1 could play a role. When the number of convolutional layers required for the temporal features was smaller than the number of

(Page 7, line 209)

MaxPool layers and AvgPool layers, changing the feature maps from the size of C×H×W to the size of C×1×1. It then went through the Share MLP module, and the parameters were shared

(Page 8, line 228-229)

As in Fig. 9, the input feature map of SAM was H×W×C. The SAM block performed maximum pooling and average pooling in one channel dimension to get two H×W×1 feature maps. Next, these two feature maps were spliced together in the channel dimension to become the H×W×2 feature map. This feature map was then passed through a convolution layer and reduced to H×W×1. After that, it passed the sigmoid function. This process can be represented as Eq. 5.

(Page 9, line 234-238)

where $\boldsymbol{F}^s_{avg}$ and $\boldsymbol{F}^s_{avg}$ denote average-pooled features and max-pooled features across the channel, and $f^{7\times7}$ represents a convolution operation with the filter size of 7×7.

(Page 9, line 239-240)

Comment 2: The matrix in Equation (4) should be bold, and there are similar errors elsewhere.

Response: Both Eq. 4 and Eq. 5 had this issue, and we have made the necessary modifications. The specific changes are as follows:

$$\begin{aligned} M_c(\boldsymbol{F}) &= \sigma(MLP(AvgPool(\boldsymbol{F})) + MLP(MaxPool(\boldsymbol{F}))) \\ &= \sigma(W_1(W_0(\boldsymbol{F}^c_{avg})) + W_1(W_0(\boldsymbol{F}^c_{max}))), \end{aligned} \quad (4)$$

$$\begin{aligned} M_s(\boldsymbol{F}) &= \sigma(f^{7\times7}([AvgPool(\boldsymbol{F}); MaxPool(\boldsymbol{F})])) \\ &= \sigma(f^{7\times7}([\boldsymbol{F}^s_{avg}; \boldsymbol{F}^s_{max}])), \end{aligned} \quad (5)$$

(Page 8, Eq. 4 and Page 9, Eq. 5)

Comment 3: The variable N in Fig. 10 needs to be represented in italics. The author needs to check all figures to ensure that all variables in the figures are also italicized.

Response: Thank you for bringing up this issue in the review. We have addressed the problem in Figures 8, 9, and 10 and have completed the necessary modifications.

We also provided the related explanations in the revised manuscript:

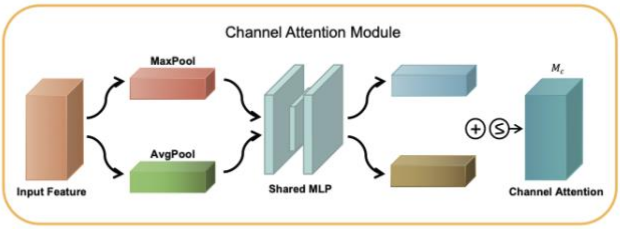

Fig 8. Schematic diagram of the CAM module structure

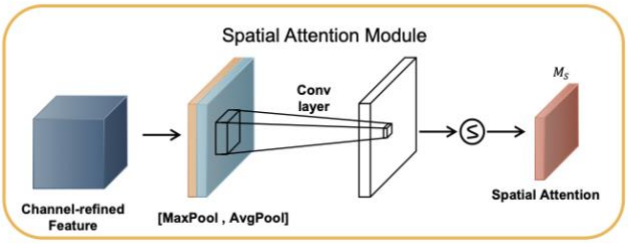

Fig 9. Schematic diagram of the SAM module structure

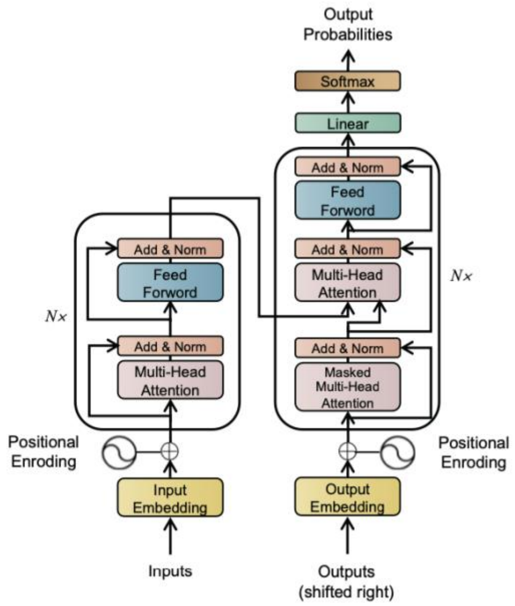

Fig 10. Schematic diagram of the Transformer structure

(Page 8, Fig 8, Page 9, Fig 9 and Page 10, Fig 10)

Comment 4: Check variables in line 352 of the manuscript.

Response: Thank you for pointing out this issue in the review. We have made the necessary modifications, and all 5 occurrences of this problem throughout the manuscript have been corrected.

We also provided the related explanations in the revised manuscript:

where b is larger than a and p denotes the percentage decrease of a relative to b.

(Page 16, Eq 12)

Comment 5: The introduction should not only be the arrangement of literature, the authors need to explain the development process of this research in detail, including the shortcomings of previous methods and the improvement of existing methods.

Response: Thank you for raising this question. We have added information in all references regarding how each referenced study improved upon previous work. These modifications have been highlighted in red font.

We also provided the related explanations in the revised manuscript:

and optimized the prediction results using an optimization function [4]. This research resolved the issue of frequent base station handovers faced by previous studies, due to inaccurate network traffic predictions. Madan et al. used the ARIMA model to predict computer network traffic and also verified that the ARIMA algorithm works mainly for time series of a linear nature [5]. This paper addressed the unresolved issue of accurately forecasting computer network traffic, accommodating both linear and nonlinear time series data, to improve quality of service (QoS) and reduce costs in data centers. However, these types of mathematical statistics-based methods are linear methods that cannot capture the nonlinear and unstable features in network traffic prediction tasks. However, most time-series forecasting tasks are subject to instabilities, such as sudden fluctuations in network traffic due to holidays and unexpected events.

There have also been many attempts to solve the task of network traffic prediction using machine learning. Jain et al. used eXtreme Gradient Boosting (XGBoost) to predict network traffic, which could provide the best solution by capturing the potential features of network traffic prediction from time-series data [6]. Compared to the previous work, this paper addressed the challenges of forecasting network traffic with irregular and abnormal data patterns as well as small cyclical data by effectively combining the Prophet and XGBoost algorithms. Li et al. presented a novel SSVM model that resolves the previously unaddressed issue of nonstationary video traffic prediction in B5G networks by accurately handling all frame types, outperforming existing models in performance. [7]. Such machine-learning algorithm-based methods have transformed the time-series problem of network traffic prediction into a supervised learning problem that can better handle more complex issues, such as the fitting of nonlinear relationships.

Recently, deep-learning algorithms have shown better results on many tasks [8]. Moreover, their ability to extract high-dimensional nonlinear features and handle large-scale data was relatively good [9]. It is their ability to handle high-dimensional nonlinear data that has led to the application of deep learning to time-series prediction tasks. Jiang addressed the previously unexplored issue of comprehensively evaluating deep neural networks for Internet traffic prediction, demonstrating that these networks outperform baseline models, with InceptionTime achieving the lowest prediction error in terms of RMSE and MAE [10]. Wan et al. tackled the previously unresolved challenge of limited sample size in network traffic prediction by introducing a novel LSTM-based neural network model with transfer learning [11]. The proposed model outperforms direct training models, achieving a performance improvement of over 40% in network traffic prediction tasks. Ramakrishnan et al. addressed the previously unexplored challenge of using Recurrent Neural Network (RNN) architectures for network traffic prediction in three domains: volume prediction, packet protocol prediction, and packet distribution prediction. The proposed RNN models, including standard RNN, LSTM, and GRU, significantly outperform standard statistical forecasting models, demonstrating their potential in various network traffic prediction applications [12]. It is not common to use Transformer for predicting network traffic, but there are quite a few works using it for similar tasks, such as predicting traffic flow. Liu et al. proposed the STGHTN for traffic flow forecasting. Compared to previous work, the difficulty of predicting complex spatiotemporal data has been reduced. Experiments confirmed its improved prediction accuracy [13]. Pu et al. introduced the MVSTT network for traffic flow prediction, effectively learning complex spatial-temporal correlations from multiple views, providing a comprehensive understanding of traffic flow dynamics. Compared to the work of predecessors, this model has significantly improved accuracy [14]. Huo et al. developed a hierarchical traffic flow forecasting network by merging LTT and STGC to address limitations in previous GCN-based methods. Compared to previous work, this model captures both short-term and long-term temporal relations in traffic flow data, while mitigating the over-smoothing problem [15].

(Page 2, Line 44-92)

Comment 6: The development process of attention should be involved.

Response: Thank you for raising this question. We have added a section in the article that describes the development process of the attention mechanism. These modifications have been highlighted in red font.

We also provided the related explanations in the revised manuscript:

> Over the years, research in natural language processing and deep learning has witnessed substantial progress in sequence-to-sequence (seq2seq) modeling, attention mechanisms, and self-attention. Initially, Sutskever et al. introduced the seq2seq model, LSTM in 2014 [16]. The seq2seq model, composed of an encoder-decoder framework, significantly improved the performance of machine translation tasks. However, the model faced limitations in handling long-range dependencies and capturing complex relationships among words in the input and output sequences. To address these challenges, Bahdanau et al. proposed the attention mechanism in 2014 [17]. The attention mechanism allowed the model to weigh the input tokens differently and focus on relevant information during the decoding process. As a result, the model demonstrated improved performance, particularly in capturing long-range dependencies and handling sentences of varying lengths. Further advancements came with the introduction of self-attention and the Transformer architecture by Vaswani et al. in 2017 [18]. The authors replaced the recurrent neural network (RNN) components in seq2seq models with the self-attention mechanism, which enabled parallel processing and efficient learning of long-range dependencies. The Transformer architecture outperformed previous models in various natural language processing tasks, including machine translation and language understanding, by effectively capturing complex word relationships in input and output sequences. Although [illegible]

(Page 2, Line 93-109)

Comment 7: The latest relevant methods based on Transformer should be mentioned.

Response: Thank you for raising this question. Indeed, it is necessary to introduce the relevant methods of the Transformer. However, the use of Transformer methods in network traffic prediction is still relatively limited. Therefore, we introduced several typical traffic flow prediction models that use the Transformer. Traffic flow prediction has some similarities to network traffic prediction. These introductions have been highlighted in red font.

We also provided the related explanations in the revised manuscript:

> [illegible] various network traffic prediction applications [12]. It is not common to use Transformer for predicting network traffic, but there are quite a few works using it for similar tasks, such as predicting traffic flow. Liu et al. proposed the STGHTN for traffic flow forecasting. Compared to previous work, the difficulty of predicting complex spatiotemporal data has been reduced. Experiments confirmed its improved prediction accuracy [13]. Pu et al. introduced the MVSTT network for traffic flow prediction, effectively learning complex spatial-temporal correlations from multiple views, providing a comprehensive understanding of traffic flow dynamics. Compared to the work of predecessors, this model has significantly improved accuracy [14]. Huo et al. developed a hierarchical traffic flow forecasting network by merging LTT and STGC to address limitations in previous GCN-based methods. Compared to previous work, this model captures both short-term and long-term temporal relations in traffic flow data, while mitigating the over-smoothing problem [15].

(Page 2, Line 81-92)

Comment 8: The authors should add variables to the structure figure of the proposed model to improve readability.

Response: Thank you for raising this question. We have added variable information in Fig 12 and included a paragraph below the image to introduce the variables. These modifications have been highlighted in red font.

We also provided the related explanations in the revised manuscript:

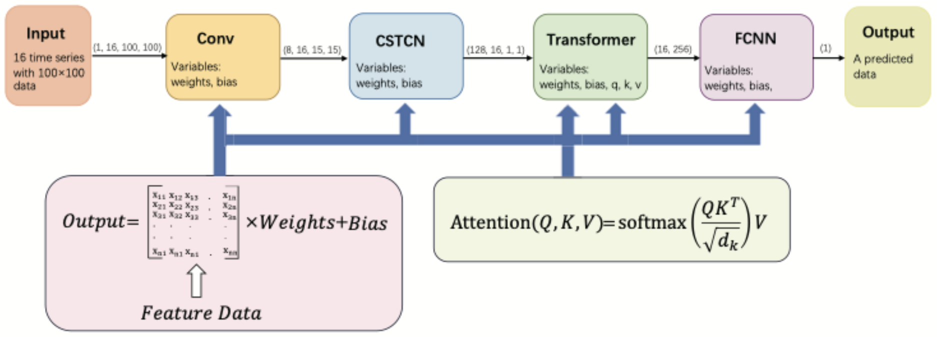

Fig 12. Overall structure of the CSTCN-Transformer

In Fig. 12, weights and bias refer to the weight and bias values of a fully connected layer or convolutional layer. Variables q, k and v respectively refer to the Query, Key, and Value in the Transformer. The tuple above the arrow indicates the shape of the current data.

(Page 11, Line 298-300)

Comment 9: The authors should explain why Pearson correlation coefficients are used instead of other correlation methods.

Response: Thank you for raising this question. Before using Pearson correlation analysis, we verified the prerequisites of this method, including that the variables follow a linear relationship, a normal distribution, and no outliers exist. Although we did not explicitly state these verifications in the article, we have added them now and highlighted these modifications in red font.

We also provided the related explanations in the revised manuscript:

time series [22]. After confirming that the data exhibits a linear relationship with minimal outliers and follows a continuous normal distribution, we used the most suitable Pearson correlation analysis for this data to investigate the correlation between network traffic data from different regions. By calculating the correlation between the network traffic of region No. 4456 and other regions, we found that the average value of the Pearson correlation coefficient between this region and other regions was just above 0.6, indicating a strong correlation. The specific

(Page 4, Line 158-163)

Comment 10: The authors need to add dimensional changes to the data in Fig. 12 to enhance readability.

Response: Thank you for raising this question. We have added the shapes of the data used in the model in Fig 12. The tuples above the arrows represent the shapes of the data. We also explained the data below the image using text.

We also provided the related explanations in the revised manuscript:

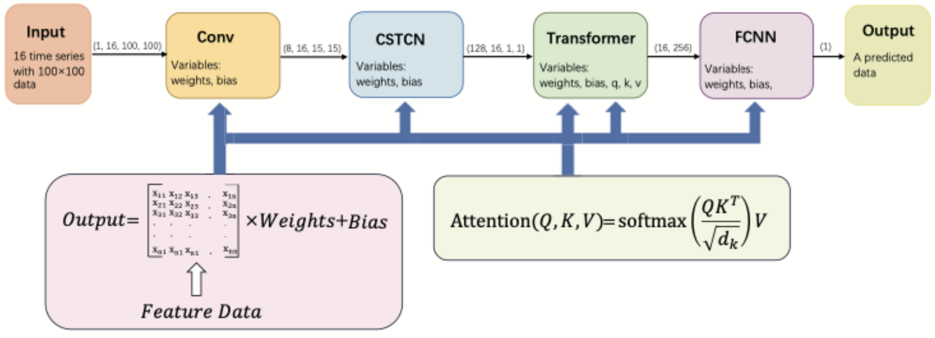

Fig 12. Overall structure of the CSTCN-Transformer

In Fig. 12, weights and bias refer to the weight and bias values of a fully connected layer or convolutional layer. Variables q, k and v respectively refer to the Query, Key, and Value in the Transformer. The tuple above the arrow indicates the shape of the current data.

(Page 11, Line 298-300)
